# Electronic Immunization Registries in Tanzania and Zambia: Shaping a Minimum Viable Product for Scaled Solutions

**DOI:** 10.3389/fpubh.2019.00218

**Published:** 2019-08-07

**Authors:** Dawn Seymour, Laurie Werner, Francis Dien Mwansa, Ngwegwe Bulula, Henry Mwanyika, Mandy Dube, Brian Taliesin, Dykki Settle

**Affiliations:** ^1^BID Initiative, PATH, Lusaka, Zambia; ^2^BID Initiative, PATH, Seattle, WA, United States; ^3^Ministry of Health, National Expanded Programme on Immunization, Lusaka, Zambia; ^4^Ministry of Health, Community Development, Gender, Elderly and Children, Dar es Salaam, Tanzania; ^5^Regional Digital Health Director–Africa, PATH, Dar es Salaam, Tanzania; ^6^Data for Action, PATH, Seattle, WA, United States; ^7^Center of Digital and Data Excellence, PATH, Seattle, WA, United States

**Keywords:** immunization, register, registry, digital, patient data, electronic immunization registry, requirements

## Abstract

As part of the work the Better Immunization Data (BID) Initiative undertook starting in 2013 to improve countries' collection, quality, and use of immunization data, PATH partnered with countries to identify the critical requirements for an electronic immunization registry (EIR). An EIR became the core intervention to address the data challenges that countries faced but also presented complexities during the development process to ensure that it met the core needs of the users. The work began with collecting common system requirements from 10 sub-Saharan African countries; these requirements represented the countries' vision of an ideal system to track individual child vaccination schedules and elements of supply chain. Through iterative development processes in both Tanzania and Zambia, the common requirements were modified and adapted to better fit the country contexts and users' needs, as well as to be developed with the technology available at the time. This process happened across four different software platforms. This paper outlines the process undertaken and analyzes similarities and differences across the iterations of the EIR in both countries, culminating in the development of a registry in Zambia that includes the most critical aspects required for initially deploying the registry and embodies what could be considered the minimum viable product for an EIR.

## Introduction

Led by PATH and funded by the Bill & Melinda Gates Foundation, the Better Immunization Data (BID) Initiative is grounded in the belief that better data plus better decisions lead to better health outcomes. The Initiative was designed in partnership with countries to create an environment where reliable, easily accessed, and actionable data can be used to improve health service delivery.

The BID Initiative has partnered with two countries, Tanzania and Zambia, over 4 years to develop, test, and roll out a package of data quality and use interventions. The governments of Tanzania and Zambia identified the most critical data-related challenges with immunization service delivery in each country, many of which were shared challenges:

- incomplete or untimely data
- inaccurate or uncertain target population for calculating immunization rates
- difficulty of uniquely identifying infants who do not start immunization or who drop out (defaulter tracing)
- lack of unique identifiers for infants
- poor data visibility at the facility level to district-level data and stock
- complex data collection forms and tools
- insufficient supply chains and logistics management
- inadequate capacity across the health system for data management and use.

Both countries formed user advisory groups made up of health workers from all levels of the health system (including community health workers, facility staff, district and provincial managers, and national-level staff) to develop a suite of interventions to address these data-related challenges. An iterative, evolutionary approach to developing the solutions was taken, building on existing systems when possible. Although interventions were designed and tested in the regions identified for initial implementation, close collaboration with government employees and agencies at the national-level focused on creating solutions that would be sustainable, that could scale beyond the initial regions, and that could be used in multiple countries with little additional development effort. Among the solutions in the suite of interventions, the most intricate to develop was the electronic immunization registry (EIR), which gives health workers access to immunization data that can be used for decision-making to improve the effectiveness and efficiency of delivering immunization services.

electronic immunization registries (EIRs) have been used in high-income countries for many years and over the past 10 years or so the movement has grown to introduce them in low and middle-income countries. EIR have been shown to improve the quality (1–3), availability, and accessibility to routine immunization data and reporting (4, 5), and provide critical information to help strengthen program performance such as identifying defaulters, increase coverage rates and timeliness of vaccination (6), and help improve stock management. As mobile technology has improved, the ability to extend such systems to low-resource settings has become a reality (7). This growing body of experiences and knowledge led to the decision to incorporate an EIR into the suite of interventions to address the challenges being faced by the two countries.

Although it was not the original intent, the requirements documented by Tanzania and Zambia for the EIR have proven to be on the cutting edge of technology for electronic immunization registries. Initially, it was assumed that existing systems would be readily found that would meet the requirements for an immunization registry. However, this was not the case and the Initiative invested in four different software platforms in the work with Tanzania and Zambia to finally arrive at the two solutions now used in both countries. The software-development work done across the four different platforms has allowed a comparison of the shared and unique requirements between countries, as well as the minimum set of requirements needed to have a usable EIR.

Throughout this process, several best practices were identified, and lessons learned were documented in planning for technology development, working with software developers and country ministries of health, and implementing new technologies successfully. These experiences can serve as a valuable resource for other countries that want to introduce an electronic immunization (or other health service) registry.

## Methods

In October 2013, the BID Initiative brought together representatives from the ICT and immunization departments of 10 sub-Saharan African countries (including Tanzania and Zambia) to develop a common set of requirements addressing their shared immunization challenges. To inform this process, the Collaborative Requirements Development Methodology (CRDM) was applied (8). The CRDM, created in 2009 by the Public Health Informatics Institute in conjunction with PATH, is used to collect and document business-process workflows and define requirements for the information systems that support those workflows. The initial set of requirements for a national EIR were compiled in the Product Vision for the BID Initiative in 2013 (9).

These documented requirements showed that countries had a forward-looking vision, especially when compared to the functionality of open source systems available at the time. The requirements demonstrated a desire for solutions that could be easily used and adapted in places where there are challenges with infrastructure, Internet connectivity, and electricity, for example. Solutions should function on mobile devices, such as tablets and phones, and meet best practices in data security and privacy. This vision represented a shift from desktop and laptop devices, which constrained nurses to desks and data entry as a separate process, to mobile devices that enabled nurses to collect data while they were performing other immunization processes and use the data on the devices to make decisions. Multiple nurses could also access and use a single shared device, and ultimately the system would be interoperable with other existing systems in the country (especially the national-level health information management system).

These initial requirements were grouped into functional and system requirements. Functional requirements describe *what* the system should do, and system requirements describe *how* the system should perform. Examples of functional requirements include registering a new child, searching for a child already registered in the system, and printing reports. System requirements include functionality such as audit logs, data backups, and user-password recovery.

Tanzania used the common requirements collected from the 10 countries in the Product Vision for the BID Initiative to develop country-specific requirements for its EIR in 2014, with the involvement of immunization, ICT, and monitoring and evaluation staff from the Ministry of Health. The Tanzania EIR requirements were then shared in a request for proposal for the software development. Tanzania initially selected the Generic Immunization Information System (GIIS) platform for its EIR, which became known as the Tanzania Immunization Information System (TIIS).

Challenges with the system led to a revised set of requirements based on lessons learned and user feedback in Arusha region where the system was tested and piloted, and a subsequent search for a different platform in 2015. Challenges encountered included synchronizing data between the two devices used in the same facility as well as with the central database, design decisions that increased the cost and ease of maintaining the source code, and projected cost of extending and replicating the system to other countries. Only the Arusha region of Tanzania continued to use TIIS once improvements were made for several more months before making the transition to a new system was underway.

In 2016, a new platform, Open Immunize (OpenIZ), was selected that would address the modified list of requirements that emerged from lessons learned with TIIS. This second system is called the Tanzania Immunization Registry (TImR) (10). TImR was used in three additional regions of Tanzania during the grant period and later replaced TIIS in Arusha region.

**Figure 1:**
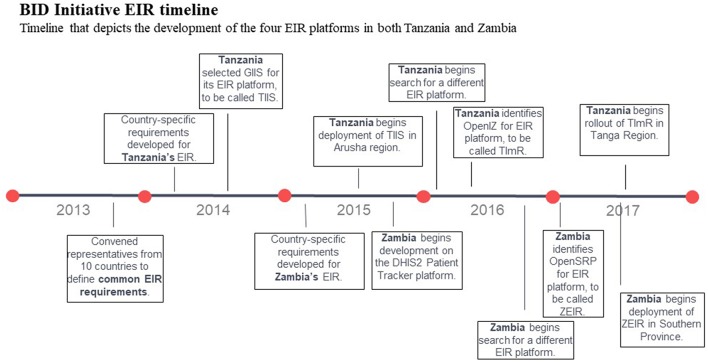
BID Initiative EIR timeline. Timeline that depicts the development of the four EIR platforms in both Tanzania and Zambia.

Zambia completed the CRDM process to develop and document country-specific requirements for its EIR (with similar involvement of stakeholders from across the ministry), and the first registry began development in 2015 on the District Health Information Software (DHIS2) Patient Tracker platform using the Patient Tracker application. Challenges adapting the software to meet some of the cutting-edge requirements, such as functionality on Android devices, ability to access and update records in offline mode, and support for multiple users per device led to stopping developing on this platform in 2016 (11). As in Tanzania, Zambia refined the requirements for their EIR based on lessons learned to a minimal set that could be adapted and deployed quickly. In 2017, Zambia selected the Open Smart Registry Platform (OpenSRP) for the second version of their EIR, which was called the Zambia Electronic Immunization Registry (ZEIR) (12) (see Figure 1 for full timeline).

## Results

The initial Product Vision requirements were refined to a modified set during the CRDM process for the first versions of the EIR in each country and further refined in the second round of development. In this iterative manner, systems were developed focusing on practical operations that would meet key workflows in the immunization process.

Tanzania has deployed the second version of its EIR, the TImR, in four regions (Arusha, Tanga, Kilimanjaro, and Dodoma) covering 1,273 facilities, and deployment to remaining regions is slated for 2019–2020. Zambia has deployed the second version of its EIR, ZEIR, in 291 facilities in Southern Province and is actively seeking funds to support maturing its use in the province, as well as scaling to other provinces.

The 5 years of experience developing EIRs in Tanzania and Zambia under the BID Initiative contributed to important lessons in system development, which were documented and disseminated (13, 14). These lessons include how to work effectively with partners, how to create a culture of data use, the importance of change management to support the transition to and adoption of a new system, and how to train health care workers efficiently in using the new tools. Specific to the EIRs, countries were forward-thinking in envisioning the technical functionality necessary to make the registry a successful tool in improving data quality and use. Through the development process, this advanced set of requirements was narrowed to those most critical for the successful initial deployment of an EIR.

In Tanzania and Zambia, the requirements were modified to reflect the collective input of immunization stakeholders across the entire health system. The modified requirements across the EIRs fell into five thematic areas of functionality in immunization service delivery (see Table 1).

**Table 1:** Common functionality groupings of requirements across EIRs.

**System**	**Number of requirements by functionality**				
	**Registration and search**	**Vaccine administration**	**Client management**	**Stock management**	**Reports**
TIIS (Tanzania)	21	18	16	19	37
TImR (Tanzania)	22	26	10	14	27
Patient Tracker (Zambia)	21	18	17	20	36
ZEIR (Zambia)	18	15	13	6	15

### Registration and Search Requirements

This theme includes uniquely identifying each child to ensure that the right child receives the right dose of a vaccine at the right time. Unique identification also enables a country to identify a true denominator of the target population that needs routine vaccines and forecast and plan for the appropriate level of stock. Other examples of registration and search functionality include registering a child in a maternity ward or immunization clinic with minimal information (e.g., no given name yet); entering the mother's or caregiver's information, including telephone number and residence; searching with partial information; and searching for a child using a bar code.

### Vaccine Administration Requirements

One key user request was the ability to display the immunization schedule of each child. Vaccine administration includes requirements for generating initial vaccine schedules based on the child's date of birth, as well as identifying children who are due and overdue for vaccines and allowing an authorized user to set the immunization schedule at the national level.

### Client Management Requirements

Client management covers the ability of the registry to identify and consolidate duplicate records and to warn if a child with the same given name, last name, date of birth, and gender already exists in the system. This functionality complements the unique identification of each child, so that nurses can validate that the right child is receiving the right vaccine dose. It also improves forecasting by providing an accurate number of children in the catchment area and birth cohort. This functionality enables the user to update client information, such as entering the child's name or the mother's mobile number.

### Stock Management Requirements

The EIRs enable health facilities to update and view their vaccine stock data. Stock management requirements include adjusting the stock balance based on a number of reasons including expiration dates, breakage, or doses reported in the EIR, alerting when stock levels are low or have expired, and aggregating vaccine-consumption tracking in terms of doses per vaccine type per time period at the service delivery point.

### Reports

Reports are needed for monitoring the performance of service delivery from the facility to the national level. Since many reports can be generated from individual records, data can be analyzed in multiple ways. Reports need to show vaccination coverage as the percentage of children living in a certain area who were born in a certain timeframe and were vaccinated with a certain dose. Reports should also categorize defaulter information by location and community health worker and report cases of adverse events. Ideally, reports are both automated and simplified, and they are submitted electronically to the district on a monthly basis.

### Minimum Viable Product

As the two countries' requirements were mapped across the four platforms, similarities and differences emerged between the initial Product Vision and the two rounds of system development. These similarities and differences highlighted requirements that could define the functionality of a minimum viable product (MVP) for an EIR that could be used to improve immunization service delivery, deployed to scale in a country, and adapted for use in other countries. By MVP we mean a product with just enough features to satisfy early users, meet the minimal functionalities, and to provide feedback for future releases of the product. The differences in the number of requirements between those initially defined in the Product Vision and those used for the different versions of the EIRs developed in Tanzania and Zambia are noted in Table 2. In addition, Supplementary Table A-1 outlines the requirements outlined in the RFP processes across the various platforms and in the final products of TImR and ZEIR.

**Table 2:** Total number of functional and system requirements across the Product Vision and four EIR platforms.

**Requirements source**	**Total requirements**	**Functional requirements**	**System requirements**
Product Vision	342	275	67
TIIS (Tanzania version 1)	138	125	13
TImR (Tanzania version 2)	155	125	30
Patient Tracker (Zambia version 1)	154	132	22
ZEIR (Zambia version 2)	85	78	7

In the first-round TIIS used or validated 57 requirements (16.6%) of the Product Vision requirements as needed for the Tanzania context. The Patient Tracker used or validated 55 requirements (16.1%) of the Product Vision requirements as needed for the Zambia context. Across all three (Product Vision, TIIS, and Patient Tracker), 54 requirements remained the same. Some of these included uniquely identifying every person, entering the vaccine vial monitor status, displaying availability of vaccine stock, and producing a report that identified all children due for a vaccination within the next month.

Some of the requirements for the second round of EIR development carried over from the first systems, but several were modified based on lessons learned from testing and deploying the initial EIRs in health facilities. In Tanzania, the TImR used or validated 35 requirements (10.2%) from the initial Product Vision but overlapped more with the TIIS: 55 requirements were the same between the two versions. In Zambia, the requirements for the ZEIR were compiled with CRDM, as well as from lessons learned from testing the Patient Tracker in health facilities. The ZEIR used or validated 29 requirements (8.5%) of the requirements from the Product Vision but overlapped more with Patient Tracker: 73 requirements were the same between the two systems (see Box 1 for current state of Patient Tracker).

Box 1 **Patient Tracker evolution.**

The experience with DHIS2 Tracker took place during 2016. In the 2 years since, The University of Oslo has invested significant time and energy to redesign the application based upon feedback from the larger community and a variety of projects such as the work conducted in Zambia with the BID Initiative. The new Android application (called DHIS2 Android Capture App) is fully integrated with the DHIS2 platform, and replaces not only the Tracker app, but also Event and Data Capture. Many key areas have been strengthened and new ones have been added, including improved performance, increased security features, and improved user experience. A new SDK was also developed, allowing for the creation of custom apps on top of DHIS2. The updated version of DHIS2 for Android was released in September 2018, and the SDK will be released by May 2019. You can find more information at www.dhis2.org/Android.

These comparisons showed that the Product Vision for the BID Initiative represented a larger vision for an EIR with more advanced requirements than what was ultimately deployed in both countries. Modifying the requirements throughout the development process narrowed the requirements to the most critical aspects needed for initial deployment of the EIRs. Areas where requirements were reduced or simplified were in stock management, facility management, complex system management, and complex reporting that was not country specific. These “dream” requirements can be applied to future versions of the EIR, especially as the system is used more frequently and scaled.

## Discussion

An EIR is a critical component of interventions to address data collection, storage, and use challenges in immunization service delivery, but such a system can be complex to design and deploy successfully. A comprehensive understanding of system requirements early in the design process is critical for ensuring that the EIR works well and is embraced by its intended users. These requirements provide the building blocks and define the capabilities of the system.

Both Tanzania and Zambia plan to scale their EIRs nationally and to integrate them with other health information systems in use in those countries, in particular the national-level health information management system which is DHIS2 in both countries. The BID Initiative also seeks opportunities to expand these solutions and to continue to learn from their development, including ways to integrate with solutions beyond immunization and routine data collection, such as supply-chain management. In addition, the Initiative's experiences refining the EIR system requirements can lead to more efficient registry development and deployment in countries that would like to put similar systems into use as well as expand to modules that cover health domains like maternal health.

The similarities and differences in requirements, as well as the groupings of common functionalities, were analyzed across the EIRs in use to understand what an MVP could look like for an EIR. This EIR should include the minimal functionality necessary to be used successfully in the health system, provide health workers with the data needed for decision-making, produce key reports for monitoring, and be scalable nationwide, as well as to other countries.

An MVP should also include international standards in the following areas:

- care guidelines [e.g., the published immunization schedules set by the World Health Organization (15)]
- content guidelines, or the equivalent of “fields” on a paper form [e.g., Health Level Seven clinical document architecture templates, such as the Immunization Content specification (16)]
- coding standards, or the standards that would apply to a specific field on a paper form [e.g., the child's sex according to the International Organization for Standardization's ISO 5218:2004 specification: 0 = unknown, 1 = male, 2 = female, and 9 = not applicable (17)]
- interoperability standards governing exchange between information systems [e.g., IHE's cross-enterprise document-sharing XDS specification (18)]
- privacy standards for personal health information [e.g., health-specific profiles such as the IHE Basic Patient Privacy and Consents specification (19)]
- security standards (e.g., cross-industry standards for authentication, encryption, and secure communication).

The process undergone in Zambia to arrive at the concise minimum set of requirements for the development of the ZEIR on the OpenSRP platform outlines what could be considered an MVP. The experience in Zambia leads to the conclusion that the 85 requirements defined for ZEIR can meet that definition of an MVP for an EIR (see Supplementary Table A-2 for the full set of requirements). The International Training and Education Centre for Health in Kenya has since adopted and adapted this system, and many other countries and implementing partners have demonstrated and shared it.

## Data Availability

All datasets generated for this study are included in the manuscript and/or the Supplementary Files.

## Author Contributions

DSey, LW, HM, MD, and DSet contributed to the design. DSey conducted the analysis and interpretation. DSey and LW wrote the first draft. HM and MD wrote additional sections. All authors contributed to manuscript revision and approved the final version.

### Conflict of Interest Statement

The authors declare that the research was conducted in the absence of any commercial or financial relationships that could be construed as a potential conflict of interest.
