# Supplementary material for: Electronic Immunization Registries in Tanzania and Zambia: Shaping a Minimum Viable Product for Scaled Solutions
Source: Front Public Health. 2019 Aug 7;7:218. doi: 10.3389/fpubh.2019.00218 (PMC6693385; doi:10.3389/fpubh.2019.00218)
Supplement: Supplementary file 1 [file Data_Sheet_1.docx]

Supplementary Material

## Supplementary Tables

**Supplementary Table A-1: Requirements shared across multiple documented sources.**

| Requirement | Product Vision | TIIS (Tanzania) | TImR (Tanzania) | Patient Tracker (Zambia) | ZEIR (Zambia) |
| --- | --- | --- | --- | --- | --- |
| Search and match on partial information (such as partial birthdates). | Yes | Yes | Yes | Yes | Yes |
| Allow the user to find patient records using bar codes/QR codes. | Yes | Yes | Yes | Yes | Yes |
| Allow the user to search for the patient given some demographic information. | Yes | Yes | Yes | Yes | Yes |
| Allow the user to select the place of birth from a list as defined by the system administrator. | Yes | Yes | Yes | Yes | Yes |
| Categorize defaulter information by location and community health worker. | Yes | Yes | Yes | Yes | Yes |
| Enter contact information (e.g., mother’s or caregiver’s mobile phone). | Yes | Yes | Yes | Yes | Yes |
| Automatically identify existing child records as duplicates. | Yes | Yes | Yes | Yes | Yes |
| Allow the user to edit, update, and override child information, such as change of address (moved permanently or temporarily). | Yes | Yes | Yes | Yes | Yes |
| Identify all children due (or overdue) for vaccination by the next clinic date and sort the list by antigen. | Yes | Yes | Yes | Yes | Yes |
| Allow the administrator to establish access privileges. | Yes | Yes | Yes | Yes | Yes |
| Enable a task to be cancelled and rolled back to its previous state. | Yes | Yes | Yes | Yes | Yes |
| Enable users to work offline and synchronize data when a connection is available. | Yes | Yes | Yes | Yes | Yes |
| Print a vaccine report and requisition form. | Yes | Yes | Yes | Yes | No |
| Capture mandatory user feedback, such as stock on hand and reason for order. | Yes | Yes | Yes | Yes | Yes |
| Search for children based on family relationships or demographics. | Yes | Yes | Yes | Yes | Yes |
| Allow the user to select the health center of the child from a list as defined by the system administrator. | Yes | Yes | Yes | Yes | Yes |
| Register children at birth in the maternity ward with minimum information. | Yes | Yes | Yes | Yes | Yes |
| Display a list of children who missed their immunizations for each antigen. | Yes | Yes | Yes | Yes | Yes |
| Enforce a minimal data set to allow for new registrations. | Yes | Yes | Yes | Yes | Yes |
| Flag duplicate records that require manual review. | Yes | Yes | Yes^[[1]](#footnote-1)^ | Yes | Yes |
| Automatically identify new child records as possible duplicates. | Yes | Yes | Yes | Yes | No |
| Combine two or more duplicate records according to business rules. | Yes | Yes | No | Yes | Yes |
| Maintain the child’s preferred contact method. | Yes | Yes | Yes | Yes | Yes |
| Prompt the user that the new vaccine is a duplicate. | Yes | Yes | Yes | Yes | Yes |
| Prevent all records given an inactive or deceased status from being included in the list of children for reminder or recall. | Yes | Yes | Yes | Yes | Yes |
| Support an audit trail when event records are merged. | Yes | Yes | Yes | Yes | Yes |
| Support real-time data-entry validation and feedback to prevent data-entry errors from being recorded. | Yes | Yes | Yes | Yes | Yes |
| Trace and record changes to data taken by the system and by users (update/delete/add). | Yes | Yes | Yes | Yes | Yes |
| Uniquely identify every person. | Yes | Yes | YES | Yes | Yes |
| Update the patient’s vaccination record with all relevant information (date, dose, lot, and number antigen). | Yes | Yes | Yes | Yes | Yes |

**Supplementary Table A-2: Requirements for a minimum viable product for an electronic immunization registry.**

| Number | Requirement |
| --- | --- |
| 1 | Register a child at birth in the maternity ward with minimum information. |
| 2 | Warn if a child is being registered with the same given name, last name, date of birth, and gender as an already registered child. |
| 3 | Warn if the identification being assigned is already assigned to another child. |
| 4–13 | Enter personal identification data into the system:   1. first name (optional) 2. family name (required) 3. mother’s name (required) 4. caregiver’s name (required) 5. national ID, such as birth certificate (optional) 6. immunization ID/QR code (required) 7. gender (required) 8. date of birth (required) 9. contact information (e.g., mother’s or caregiver’s mobile phone). |
| 14 | Allow the user to select the place of birth from a list as defined by the system administrator. |
| 15 | Enforce field validation to refer to a reference table of prepopulated values (e.g., villages within health-facility capture radius or within the country). |
| 16 | Define communities, places of birth, places of domicile, coverage areas, and any dependencies between them. |
| 17 | Allow the user to select the health center of the child from a list as defined by the system administrator. |
| 18 | Allow the user to search for the patient given some demographic information. |
| 19 | Maintain the status of a child record; this status field will be used for reporting and vaccination planning in other areas of the system. |
| 20 | Define which vaccines are offered by the national immunization program and at what age the respective doses are recommended. |
| 21 | Identify all children due (or overdue) for vaccination by the next clinic date and sort the list by antigen. |
| 22 | Monitor the performance of the immunization program by comparing the number of immunized children to the number of children that live in the program area. |
| 23–26 | Define the ideal age within the national vaccine schedule with the following three constraints; should   1. the minimum age before which a child is not eligible for a certain dose 2. the maximum age beyond which a child is not eligible for a certain dose 3. a minimum time that needs to pass between doses of the same vaccine. |
| 27 | In the event a provider overrides a warning message to provide a vaccine out of the recommended range, require that a reason be given (based on a dropdown list of choices or a free-text “other” field). |
| 28 | Update the patient’s vaccination record with all relevant information (date, dose, lot number, and antigen). |
| 29 | Reproduce (display and print) a child’s vaccination history, together with all due and overdue appointments. |
| 30 | List vaccinations done and not done for a child according to schedule (vaccination card). |
| 31 | Reproduce (display and print) a coverage report that shows vaccination coverage as the percentage of the children living in a certain area who were born in a certain timeframe and were vaccinated with a certain vaccine dose (cohort reporting). |
| 32 | Reproduce (display and print) a report that shows all vaccinations administered by dose and by health facility or group of health facilities (district, region, and country). |
| 33 | Maintain the child’s preferred contact method. |
| 34 | Prevent all records given an inactive or deceased status from being included in the list of children for reminder/recall. |
| 35 | Edit, update, and override child information, such as change of address (moved permanently or temporarily). |
| 36 | Display a list of children who missed their immunizations for each antigen. |
| 37 | Allow the user to assign a village or subvillage to a child. |
| 38 | Categorize defaulter information by location and community health worker. |
| 39 | Search and match on partial information (such as partial birthdates). |
| 40 | Search for children based on family relationships or demographics. |
| 41 | Allow the user to find patient records using bar codes. |
| 42 | Enforce a minimal data set to allow for a new registration. |
| 43 | Uniquely identify every person. |
| 44 | Have ability to automatically identify new child records as possible duplicates.^[[2]](#footnote-2)^ |
| 45 | Have ability to automatically identify existing child records as duplicates. |
| 46 | Flag duplicate records that require manual review. |
| 47 | Have ability to combine two or more duplicate records according to business rules. (Note: business rules should define which criteria to use to merge records, such as the information to keep from the duplicates). |
| 48 | Allow a record to have multiple alternate IDs. |
| 49 | When records are merged or combined, maintain a reference to the previous (or nonsurviving) ID that could be found on search. |
| 50 | Have ability to prompt the user that the new vaccine is a duplicate. |
| 51 | Have ability to combine two or more duplicate event records according to business rules. |
| 52 | Support an audit trail when event records are merged. |
| 53 | Support real-time data-entry validation and feedback to prevent data-entry errors from being recorded. |
| 54 | Support synchronization of data between a facility device and the national system. |
| 55 | Trace and record changes to data taken by the system and by users (update/delete/add). |
| 56 | Have the ability to record immunizations from children who are from other villages. |
| 57 | Have the ability to record that an immunization was given during an outreach campaign. |
| 58 | Maintain a list of vaccines and other drugs that are provided in the country. |
| 59 | Generate dashboards. |
| 60 | Generate a Health Information Aggregation form 2 report monthly, which should be both printable and automatically imported into DHIS2. |
| 61 | Aggregate vaccine consumption tracked, in terms of doses of vaccine type per time period (e.g., doses of Bacille Calmette-Guerin (BCG) vaccine consumed since May 21) at the SDP level. |
| 62 | Maintain default reorder point and replenishment quantities by vaccine type by stock point (facility). |
| 63 | Submit aggregate data to calculate coverage rate. |
| 64 | Allow user to choose a report-generation time frame (i.e., run now or set the time for later). |
| 65 | Have ability to validate vaccine availability and update stock counts. |
| 66 | Have ability to alert on low/expired stock level. |
| 67 | Have ability to adjust stock balance based on given vaccines/doses report in immunization registry. |
| 68 | Have ability to display stock balance/details in offline mode by downloading the stock information as a last synchronization, adjust stock information while in offline mode based on immunization registry transactions, and provide alerts on stock balances based on reorder, minimum, maximum, or buffer levels. |
| 69 | Provide a snapshot of what is happening when the user first logs in to the system. |
| 70 | Calculate dropout rate trend. |
| 71 | Have ability to report cases or adverse events. |
| 72 | Have ability to enter historical data during the patient’s first visit to the health center (e.g., doses given at birth). |
| 73 | Do not remove data from the device when a unique user logs out of the system. |
| 74 | Enable a unique user to log in again to the system when the Internet connection is lost or unavailable. |
| 75 | Implement support for multiple users per device with user-switching functionality. |
| 76 | Implement support for discarding events. |
| 77 | Provide patients with their own paper immunization records. |
| 78 | Change storing approach. |
| 79 | Produce vaccinated children report. |
| 80 | Produce stock on hand report. |
| 81 | Produce stock on hand versus demand report. |

1. This functionality does occur with TImR but is done by the client registry and is not a core functionality of TImR. [↑](#footnote-ref-1)
2. While this functionality was not in ZEIR initially, based on our experience with client management being able to prevent duplicate records will help with more accurate data and decision-making and therefore have left it in the list. [↑](#footnote-ref-2)
